# Implementing a Community-Based Collaborative Project During the COVID-19 Pandemic: A Process Evaluation

**DOI:** 10.1093/geroni/igab046.2816

**Published:** 2021-12-17

**Authors:** Jarmin Yeh, Leslie Ross, Brittney Pond, Brooke Hollister, Tiffany Cheang, Susie Yu, Corinne Eldridge

**Affiliations:** 1 University of California, San Francisco, San Francisco, California, United States; 2 University of California, San Francisco, University of California, San Francisco, California, United States; 3 University of California, San Francisco, California, United States; 4 Alameda Alliance for Health, Alameda, California, United States; 5 Center for Caregiver Advancement, Los Angeles, California, United States

## Abstract

In-Home Supportive Services (IHSS) caregivers are critical linchpins in our long-term care system, but little research exists to examine the strategies for enhancing their role working in the homes of persons living with dementia (PLWD). The aim of the IHSS+ Alzheimer’s Disease and Related Dementias Training Project (IHSS+ ADRD Training Project) is to implement a competency-based dementia training program for 600 IHSS caregivers and their consumers; and evaluate the training program’s impact on caregiving, long-term services and supports, and health systems. This project is a partnership between the University of California, San Francisco, Institute for Health & Aging; Center for Caregiver Advancement, a nonprofit organization founded by home care workers; and Alameda Alliance for Health, a non-profit managed care plan created by and for residents of Alameda County, California. The research design and funding of the project was established just prior to the COVID-19 pandemic. As the pandemic evolved, the IHSS+ ADRD Training Project underwent many revisions and pivoted to remote strategies to ensure progress could be made toward the aims. This poster describes a process evaluation of how various challenges were addressed and subsequent changes were made to the methodology. Using a community-based participatory research and program evaluation hybrid model, this project remained nimble, configuring an online dementia training program and evaluation methods that accommodated safety needs of community partners. Benefits and limitations of implementing the IHSS+ ADRD Training Project, using remote strategies, to ensure IHSS caregivers and their consumers could continue receiving education and support, are highlighted.

